# Analysis of Disposed Unused Medications at a Village Community Pharmacy

**DOI:** 10.3390/pharmacy7020045

**Published:** 2019-05-12

**Authors:** Valerie Vella, Lorna-Marie West

**Affiliations:** 1 Community Pharmacist, MGR1326 L-Imgarr, Malta; 2 Department of Clinical Pharmacology and Therapeutics, University of Malta, MSD2080 Msida, Malta; lorna.west@um.edu.mt

**Keywords:** community pharmacy, cost, disposal, expiry date, medication waste

## Abstract

**Background:** The aim of this study was to determine the type, quantity, and cost of medications being disposed of by clients in a specifically-set pharmaceutical disposal bin at a village community pharmacy. **Methods:** Medicines placed in a medication disposal bin by clients were examined during a nine-month period from April to December 2018. The data recorded included the active ingredient, trade name, dose, dosage form, disposed quantity, and the actual expiry date on the pack. The medications were classified according to ATC (Anatomical Therapeutic Chemical Classification System) code, and the cost of the amount wasted was calculated using the pharmacy’s price list. Descriptive statistics were used to analyze the data. **Results:** A total of 411 medications were collected, amounting to a total cost of approximately €2600. The largest group of medications belonged to the alimentary group, and this also represented the group with the highest monetary value. The number of months that medicinal products were retained by patients beyond the expiry date ranged from 1 to 232. **Conclusion**: This small study provides a glimpse of what clients dispose of in a medication bin when this is readily available in their community pharmacy, a simple measure which, if adopted on a national level, could aid in ensuring the appropriate disposal of wasted medication.

## 1. Introduction

A systematic review of the literature, which included 42 published primary research papers, found that the majority of the studies (74%) quantified medication wastage [1], with the extent and type of wasted medication varying between studies [2,3]. The direct cost of medication waste is a major concern which can jeopardize the sustainability of healthcare systems, with some studies estimating millions of euros in wasted medication when extrapolating their findings on a national level [4,5]. A study by Law et al. [6] estimated a direct cost of $5.8 billion in terms of wasted medication for those US adults who are on at least one chronic prescription medication daily. Moreover, while there is ample evidence indicating that inappropriate medication waste disposal has a negative impact on the environment [7], studies still show that the most common method of medication waste disposal in most countries is through the normal sewage system [8].

While the extent of medication wastage has been reported for other countries, as indicated from the systematic review discussed above [1], studies physically quantifying and costing medication waste in Malta are lacking. Therefore, the rationale for the current study was to generate data on medication waste for the Maltese Islands. Moreover, a survey conducted by WasteServ in 2014 found that only a tenth of the Maltese population dispose of expired medication generated within households appropriately at one of the civic amenity sites [9,10] situated across the Maltese Islands. In line with the latter study, another survey-based study amongst the Maltese population concluded that most Maltese residents still dispose of unused medication via landfill or the domestic sewerage system [11]. Since most community pharmacies in Malta do not have specific bins for the disposal of unused or expired medication returned by patients, the feasibility of having such a bin available is still unclear. Therefore, the aim of this study was to determine the type, quantity, and cost of medications being disposed of by clients in a specifically-set pharmaceutical disposal bin at a village community pharmacy and to provide an insight on the feasibility of having this bin available within the community pharmacy.

## 2. Materials and Methods

### 2.1. Setting

The study was conducted from 1 April to 31 December 2018 within a community pharmacy in a small Maltese village with 3500 inhabitants. The village is in a rural area of the island, and the majority of the residents are Maltese. There are two pharmacies in the village, and it is estimated that the pharmacy in the study sees to approximately half of the inhabitants.

### 2.2. Study Design

This study applied a cross-sectional observational design. A medication disposal bin was placed inside the community pharmacy close to the pharmacy entrance whereby patients could dispose of their expired or unused medications. The service was not formally advertised to the 3500 inhabitants of the village. However, the local council was informed about the service set by the pharmacy, and clients were also informed and encouraged by the managing pharmacist, who is also the primary author, to make use of this facility. Any individual residing in Malta at the time of the study could attend the pharmacy to dispose of unused or expired medications. At the end of every 3-month interval the managing pharmacist would empty the pharmaceutical bin and record the active ingredient, the tradename, strength, dosage form, quantity, and expiry date of each medication.

### 2.3. Data Analysis

Data were entered in a Microsoft Excel^®^ sheet and analyzed using descriptive statistics. Solid dosage forms were counted manually, liquid dosage forms were measured using a calibrated measuring cylinder, dermatological preparations were measured using kitchen weighing scales, and inhalers which had a counter were recorded as per value available on the counter. Unused inhalers without a counter, eye drops, ear drops, nasal drops, and nasal and oral sprays were not quantified as effective entries, as their quantities could not be safely determined.

The cost of the medication returned was calculated using the retail price as per the pharmacy’s price list issued in March 2019. The two main reasons for choosing the retail price were first, that this was the cost paid by the client, and secondly, retail prices in Malta are standard across pharmacies and, unlike purchasing prices, do not vary according to special bonuses issued by pharmaceutical agents. For items for which a price could not be found, e.g., medicines purchased from abroad, the British National Formulary March 2019 [12] price was used and converted to euros using the exchange rate of March 2019 [13].

The expiry date on the pack was used to estimate the time, in months, that the medication was held beyond the expiry date at the client’s end. Items which were found in the pharmaceutical bin but were not yet expired were classified under two categories. Medicines which were not expired but had specific instructions on the packaging detailing how long they could be used after opening were classified as medicines having “specific after opening instructions”. This was the case, for example, for eye drop preparations, certain liquid preparations, and nasal sprays. On the other hand, medicines classified as “not yet expired” consisted of medications that could still be utilized at the time at which they were thrown away. When the expiry date was not visible on the pack or blister pack, this was categorized as “expiry date not visible”.

The returned medications were classified according to the World Health Organization (WHO) ATC code [14]. A few of the returns could not be classified using the ATC classification, as these were herbal remedies or food supplements. These were grouped under the sub-heading “Others”.

## 3. Results

### 3.1. Quantity

During the nine-month period, a total of 411 medications which could be identified were disposed of at the pharmacy. In addition to this, a plastic bag containing loose capsules and tablets weighing 1.2 kg was also found in the medication disposal bin. However, this was not opened and analyzed in view of health and safety reasons. The distribution of medicines disposed of during the nine-month period is demonstrated in Table 1.

### 3.2. Types of Medication

Slightly more than half (53.5%, *n* = 220) of the disposed medications were prescription-only medicines, while 46.5% (*n* = 191) were over-the-counter medications. The most common class of disposed medications was that pertaining to the alimentary tract (24.6%), closely followed by medicines belonging to the respiratory group (23.8%). As can be seen in Table 2, 10.5% of the unused disposed medications were from the musculoskeletal group, which includes medications such as Non-Steroidal Anti-Inflammatory Drugs (NSAIDs), and supplements, like glucosamine. The medications with the lowest return rate were anti-neoplastic and immunomodulating agents (0.7%), followed by anti-parasitic medications (0.2%).

### 3.3. Cost

As detailed in the methodology, the amount of certain dosage forms returned (unused quantity of inhalers without a counter, eye drops, ear drops, nasal drops, and nasal and oral sprays) was not measured and, thus, the 35 preparations with these dosage forms were excluded from the cost analysis. The cost of the contents of the pharmaceutical bin in the nine-month study period totaled €2613.28. Table 2 indicates the distribution of the costs according to the ATC classification. The cost of medicines found which were not yet expired and could still be utilized was €333.99. Medications for the alimentary tract and metabolism disorders were the class where there was the highest monetary value of waste, followed by drugs used in musculoskeletal disorders, drugs used in respiratory conditions, and anti-infective medications. The categories with the lowest cost were equivalent to the categories with the lowest quantity returned. The total monetary cost associated with the disposed prescription-only medication was €974.04, while disposed over-the-counter medication amounted to €1639.24.

### 3.4. Time beyond Expiry Date Medication was Retained before Returning for Disposal

**Figure 1:**
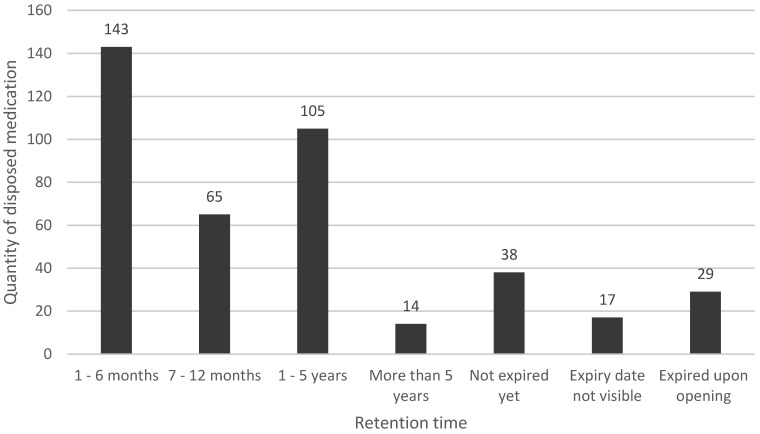
Distribution of retention time of disposed medicines beyond their expiry date.

Of the 411 disposed medications, 9.2% (*n* = 38) could still be utilized as they were not yet expired. An additional 7.1% (*n* = 29) of medications had an expiry date which was still valid but had specific instructions from the company with regards to the retention of medication once opened and, therefore, could not be utilized by clients. The majority of these preparations were eye and nasal preparations. For 17 (4.1%) medications, the expiry date could not be determined, mainly because the packaging had been tampered with. Of the remaining 327 expired preparations, the time in months which had been retained by clients before disposing of them ranged from one month to 232 months, with a mean and median of 15.5 months and 8 months respectively. Figure 1 illustrates the timeframe for each medication from the date of expiry to the date that the patient disposed of it. The majority of medications were disposed of shortly after expiry.

## 4. Discussion

### 4.1. Quantity of Disposed Medications

In Malta, the system for recycling waste in households was implemented in 2002, with the system evolving further, until in 2018, the separation of organic waste, also within households, was introduced [9]. Yet, other types of waste, such as bulky appliances, computer accessories, and pharmaceutical waste still need to be taken to one of the six civic amenity sites. However, as studies clearly indicate, Maltese residents still do not make proper use of this disposal scheme for their unused medications and instead resort to inappropriate means of disposal [11]. This could be due to the fact that when residents need to get rid of bulky refuse, they might not hesitate to take the trip to the site. However, this may be thought of as taxing when dealing with a small volume of left-over medicine. Additionally, while most patient information leaflets in medication packages suggest the return of unused or expired medication to the community pharmacy for disposal, in Malta, community pharmacists accept these at their discretion. This study has shown that a simple measure, which was not advertised through any campaign, supported individuals in conducting adequate medication disposal behaviors. This in turn, could minimize the personal and environmental risks imposed by the incorrect disposal of medications. This study also showed that, when such a service is available, the majority of individuals would possibly dispose of their medication as soon as it expires. In the absence of such a service, patients would end up retaining expired medication within their households for a longer period of time with potential safety risks for family members, such as accidental poisoning if found by children or pets. Currently, community pharmacists who hold a medication disposal bin within their pharmacy have to take the medicinal contents directly to one of the civic amenity sites themselves. A reimbursement system for community pharmacists to dispose of pharmaceutical waste may aid pharmacists in taking a more active role and make it easier and more accessible for patients to dispose of medications correctly. Alternatively, in order to support community pharmacies with this initiative, WasteServ could regularly collect the unused or expired medicines directly from pharmacies.

### 4.2. Classification of Disposed Medication

As opposed to the majority of literature which classified medication waste and found that the cardiovascular [15,16,17,18,19,20,21,22,23] and central nervous system [3,24,25,26,27,28] medications were the most commonly wasted groups, the current study found the alimentary tract group to be the most disposed of. It is not surprising that this group was found to be the one most-disposed of, since it consists not only of medications used in gastrointestinal disorders, but also medications used in diabetes, as well as vitamins and mineral supplements. In Malta, diabetes is highly prevalent, with 10% of adults known to have this condition [29]. Therefore, anti-diabetic medication may be commonly found in Maltese households. Yet, unexpectedly, this study found a low amount of disposed cardiovascular medications. A possible reason for this could be that since patients are given most cardiovascular medications free-of-charge by the Maltese National Health System (NHS), they are concerned that if they publicly dispose of these medications, their entitlement would be withdrawn, a common misconception amongst the Maltese population [30].

The second-most-common group of medications that was disposed of was the respiratory group, which was also prevalent in other studies [31,32]. The reason for a high prevalence of expired respiratory medications could be due to the fact that this group included seasonal medication, such as nasal drops and cough syrups. These medications are used mainly in the winter season and might expire before they can be reused the following year. In such instances, patients have no other option but to dispose of these expired medications. However, at times, patients might purchase different brands of the same cough syrups on different occasions without knowing that they could use the ones that they already have at home. Pharmacists should, therefore, enquire about what is already available at the patient’s home before dispensing new preparations. They should also encourage patients to make a list of the medicines that they have at home before going to their doctor.

### 4.3. Monetary Value of Disposed Medication

The direct cost of wasted medication within this study was €2613.28. This was generated over a nine-month period in a village of 3500 inhabitants. If this amount had to be extrapolated over a one-year period it would translate into approximately €3500 per year, which is one euro per inhabitant in this small village. By extrapolating this cost across the Maltese Islands, with a population of approximately 450,000 inhabitants, it would be estimated that almost €450,000 worth of medication is wasted. Yet, since the service was not advertised, few patients disposed of their unused or expired medication in the bin and, therefore, this monetary value is just the tip of the iceberg of a much larger issue. Also, the reasons as to why the medication was wasted were not explored in this study. Studies have found various reasons for medication wastage, with non-adherence being a high contributory factor and significantly associated with this phenomenon [33]. Therefore, while it is imperative to educate the public through health campaigns on how to reduce medication wastage [34], reasons for non-adherence should be addressed. A study carried out amongst patients with chronic conditions (cardiovascular, diabetes, and asthma) in Malta found that patients’ beliefs contributed significantly to non-adherence, with “concerns” about their medication being the negative driving force [33]. Community pharmacists are at the forefront of patients’ healthcare and, therefore, are in an ideal position to target patients’ concerns about their medication and consequently enhance medication adherence.

This study found that almost equal amounts of prescription-only medication and over-the-counter medication were disposed. Yet, the latter carried a higher monetary value. The group with the highest cost of expired/unused medications belonged to the alimentary tract group, probably reflecting the quantity of items returned from this group. This group included a number of over-the-counter preparations, such as vitamins and supplements, which were disposed of by patients. While patients buy vitamins and supplements over-the-counter for various reasons, the benefits of these for specific indications, if any, may not always be clear, and patients’ expectations may not be met. This could be a possible reason why patients stop taking vitamins and supplements. It is, therefore, imperative for the pharmacist to discuss with patients their reasons for purchasing these supplements and counsel them accordingly. The alimentary tract group also includes gastro-intestinal preparations, such as antacids, which are supplied in large packs. Educating the public to buy smaller packs for medication which will be used only when required is imperative. Yet, the lack of availability of smaller packs in such instances is a major limitation which can only be mitigated with the support of pharmaceutical companies.

### 4.4. Limitations of the Current Study

This study has a number of limitations which need to be acknowledged. While this study gives an indication of the existence of the medication wastage phenomenon in Malta, there are a number of methodological weaknesses which need to be addressed in future research. The owner of this community pharmacy has a particular interest in this area, so it is undeniable that her clients are indirectly encouraged to follow this good practice. The setting needs to be extended to include more than one community pharmacy in all different regions across the Maltese Islands. The number of patients disposing of unused or expired medication, together with reason for disposal, need to be recorded. This study excluded some dosage forms whilst quantifying and costing waste, such as eye drops, inhalers, and nasal sprays. Therefore, the actual cost of waste presented in this study is an underestimate.

The monetary value of the disposed quantity was not calculated according to the retail price at the time the medicine was bought and, thus, the actual cost of waste could be different owing to changing medicine prices through the years. Also, when costing medications, this study did not distinguish between medications which were bought versus those which were given to the patient for free via the Maltese NHS. Therefore, the calculated price could be different from the actual purchasing price that was paid by the Maltese NHS. In view of these limitations, this study’s findings cannot be extrapolated to the whole country.

### 4.5. Future Research

As discussed above, further research needs to be carried out in a larger sample of community pharmacies, as well as in other settings, such as hospital pharmacies and wards. Rather than just quantifying waste, this should be presented in line with the amount of medication that was supplied and reasons for wasted medication. Focus groups or interviews with clients could give a better understanding of their practices and beliefs about medication storage and disposal, and the role of the community pharmacist in all this. Future research could also explore the impact of public educational campaigns on the medication wastage phenomenon.

## 5. Conclusions

Medication wastage is a concern both for its financial and environmental impacts. This small study provides a glimpse of what clients dispose of in a medication bin when this is readily available in their community pharmacy, a simple measure which, if adopted on a national level, could aid in ensuring the appropriate disposal of wasted medication.

## Figures and Tables

**Table 1:** Distribution of disposed medication during the nine-month period.

Time Period	Number of Disposed Medications	Value of Disposed Medications (€)
April–June 2018	148	886.04
July–September 2018	71	377.04
October–December 2018	192	1350.20
**Total**	**411**	**2613.28**

**Table 2:** Quantity and cost of medications returned according to the ATC classification.

Code	Classification	Quantity Returned (*n* = 411) % (*n*)	Cost in Euros
A	Alimentary tract and metabolism	24.6% (101)	619.39
R	Respiratory System	23.8% (98)	309.93
M	Musculoskeletal system	10.5% (43)	397.82
D	Dermatologicals	8.8% (36)	208.85
J	Anti-infectives for systemic use	8.8% (36)	281.94
N	Nervous system	6.8% (28)	207.38
S	Sensory organs	3.6% (15)	51.96
C	Cardiovascular System	3.4% (14)	245.25
H	Systemic hormonal preparations	2.9% (12)	73.23
G	Genito-urnicary system and sex hormones	2.7% (11)	77.83
Others	Food supplements, herbal preparations	1.9% (8)	73.75
B	Blood and blood-forming organs	1.2% (5)	52.99
L	Antineoplastics and immunomodulating agents	0.7% (3)	10.46
P	Antiparasitic products, insecticides, and repellents	0.2% (1)	2.50
